# Photoreflectance and Photoluminescence Study of Antimony Selenide Crystals

**DOI:** 10.1021/acsaem.2c02131

**Published:** 2022-11-16

**Authors:** Rokas Kondrotas, Ramu̅nas Nedzinskas, Jüri Krustok, Maarja Grossberg, Martynas Talaikis, Saulius Tumėnas, Artu̅ras Suchodolskis, Raimundas Žaltauskas, Raimundas Sereika

**Affiliations:** † State Research Institute, Center for Physical Sciences and Technology, Saulėtekio Avenue 3, Vilnius10257, Lithuania; ‡ Department of Materials and Environmental Technology, Tallinn University of Technology, Ehitajate Tee 5, 19086Tallinn, Estonia; § Vytautas Magnus University, K. Donelaičio street 58, 44248Kaunas, Lithuania

**Keywords:** Sb_2_Se_3_ crystal, *V*_OC_-deficit, photoluminescence, photoreflectance

## Abstract

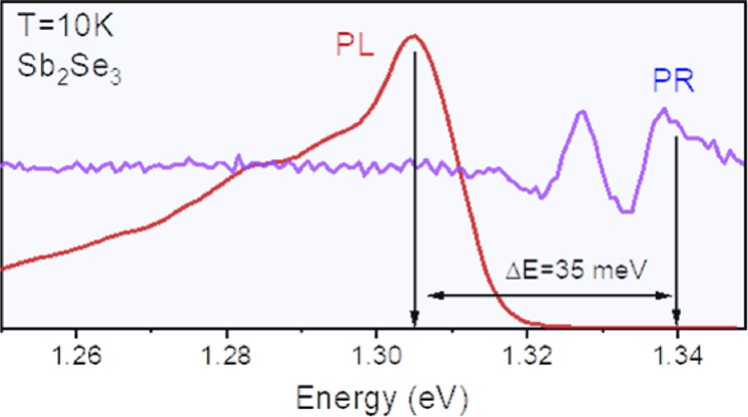

Among inorganic, Earth-abundant, and low-toxicity photovoltaic technologies, Sb_2_Se_3_ has emerged as a strong material contender reaching over 10% solar cell power conversion efficiency. Nevertheless, the bottleneck of this technology is the high deficit of open-circuit voltage (*V*_OC_) as seen in many other emerging chalcogenide technologies. Commonly, the loss of *V*_OC_ is related to the nonradiative carrier recombination through defects, but other material characteristics can also limit the achievable *V*_OC_. It has been reported that in isostructural compound Sb_2_S_3_, self-trapped excitons are readily formed leading to 0.6 eV Stokes redshift in photoluminescence (PL) and therefore significantly reducing the obtainable *V*_OC_. However, whether Sb_2_Se_3_ has the same limitations has not yet been examined. In this work, we aim to identify main radiative carrier recombination mechanisms in Sb_2_Se_3_ single crystals and estimate if there is a fundamental limit for obtainable *V*_OC_. Optical transitions in Sb_2_Se_3_ were studied by means of photoreflectance and PL spectroscopy. Temperature, excitation intensity, and polarization-dependent optical characteristics were measured and analyzed. We found that at low temperature, three distinct radiative recombination mechanisms were present and were strongly influenced by the impurities. The most intensive PL emissions were located near the band edge. In conclusion, no evidence of emission from self-trapped excitons or band-tails was observed, suggesting that there is no fundamental limitation to achieve high *V*_OC_, which is very important for further development of Sb_2_Se_3_-based solar cells.

## Introduction

In the last decade, antimony selenide (Sb_2_Se_3_) has emerged as a highly promising sustainable Earth-abundant photovoltaic (PV) material.^1^ Power conversion efficiency (PCE) of over 10% has been demonstrated for antimony chalcogenide-based solar cells.^2^ Nevertheless, the open-circuit voltage deficit (*V*_OC_-deficit) defined as a lack of photovoltage relative to the material bandgap remains the bottleneck of this PV technology. Even for the highest-efficiency solar cells, the *V*_OC_-deficit is in the 0.6–0.7 V range, that is, almost twice as much as that for well-established thin-film polycrystalline PV technologies (CdTe, CIGS).^3^ In general, a loss of the *V*_OC_ is related to the nonradiative recombination through defects or other nonradiative channels in the absorber or at interfaces.^4^ However, it can also be limited by other fundamental factors, such as band-tails,^5^ formation of self-trapped excitons,^6^ or potential fluctuations.^7^ It has been suggested that the presence of a high density of deep point defects in Sb_2_(S,Se)_3_ is the main origin of a significant *V*_OC_-deficit.^8^ Defects or their undesirable effects on charged carriers can be minimized by passivation methods,^9^ increased crystalline quality,^10^ or through cationic/anionic alloying.^11^ However, it has not been shown whether Sb_2_Se_3_ exhibits other limiting factors for *V*_OC_, such as that has been proposed for another binary antimony chalcogenide, stibnite (Sb_2_S_3_).^6^ Authors consistently found a large Stokes redshift in Sb_2_S_3_ (Figure S1). Using the time- and intensity-resolved transient absorption method, they concluded that self-trapped excitons readily formed in Sb_2_S_3_ which was also supported by theoretical calculations. Such an inherited characteristic of Sb_2_S_3_ has a significant implication on Sb_2_S_3_-based solar cells reducing theoretical efficiency from 28% to only 16%. Thus, it is important to understand fundamentals of the Sb_2_Se_3_ electronic band structure and its features that could potentially put the upper limit for obtainable *V*_OC_ and consequently PCE.

Photoluminescence (PL) is a powerful and direct emission-like spectroscopic tool to investigate the band structure and defects and also allows estimation of the degree of nonradiative recombination.^12,13^ However, PL examines usually the lowest energy optical transitions, and in order to reveal higher energy (interband/band-to-band) transitions, excitation power density is increased, which may subsequently lead to structural degradation/burning of the sample investigated. On the other hand, photoreflectance (PR) is an absorption-like, nondestructive technique, and because of its high sensitivity (the laser-induced changes in reflectance dR are on the order of 10^–4^–10^–5^ with respect to reflectance R) to probe the critical points (CPs) of the band structure, PR provides fundamental information regarding optical processes near and above the band edges.^14^ These techniques combined can directly provide a Stokes energy shift and allow for a comprehensive study of optical yield and losses in a semiconductor. However, there are only very few reports on Sb_2_Se_3_ PL properties and much less about PR. Common knowledge is that a high density of defects provides many nonradiative channels, and therefore, PL emission yield is very low, and usually absent at room temperature in thin films. In addition, Sb_2_Se_3_ is stated to be an indirect bandgap semiconductor which again leads to low PL emission, whereas for the PR study high-crystalline quality samples are required. Recently, broad defect-related PL emission was observed in Sb_2_Se_3_ microcrystals under continuous-wave (CW) excitation and biexcitonic/excitonic under high excitation density.^15,16^

However, to understand the optical properties of Sb_2_Se_3_ and, more importantly, fundamental limitations for application in solar cells, it is also necessary to study high-quality single crystals. Basic properties such as electrical conductivity, bandgap, absorption, and anisotropy of Sb_2_Se_3_ single crystals have been studied before,^17,18^ but to the best of our knowledge, there are no reports on PL or PR characteristics. Herein, we present a temperature, excitation, and polarization-dependent PR and PL study of Sb_2_Se_3_ single crystals. We found that at low temperature the PR signal consisted of three components of different origin. PL studies revealed multiple overlapping peaks of excitonic nature in addition to the defect-related band. The most dominant PL peaks were located near the band edge of Sb_2_Se_3_, leading to a small Stoke redshift, and therefore no significant photovoltage loss limiting obtainable *V*_OC_ is expected.

## Experimental Section

Sb_2_Se_3_ single crystals were grown using the Bridgman–Stockbarger technique. Precursor materials Sb (99.99% Sigma-Aldrich) and Se (99.99% Sigma-Aldrich) were weighed (total mass 20 g) and placed in the quartz ampoule (15 cm in length and 1 cm in inner diameter). Ampoule was evacuated and flame-sealed. Precursor synthesis and mixing were performed in a rolling furnace. The temperature was increased slowly up to 630 °C, that is, just above the melting point of Sb_2_Se_3_. After 24 h mixing, temperature was slowly reduced to room temperature. Then, the ampoule was placed in a vertical furnace and lowered at a rate of 1.05 mm/h through the hot zone. The maximum temperature in the center of the vertical furnace was 620 °C. This process took 1 week to complete.

**Figure 1:**
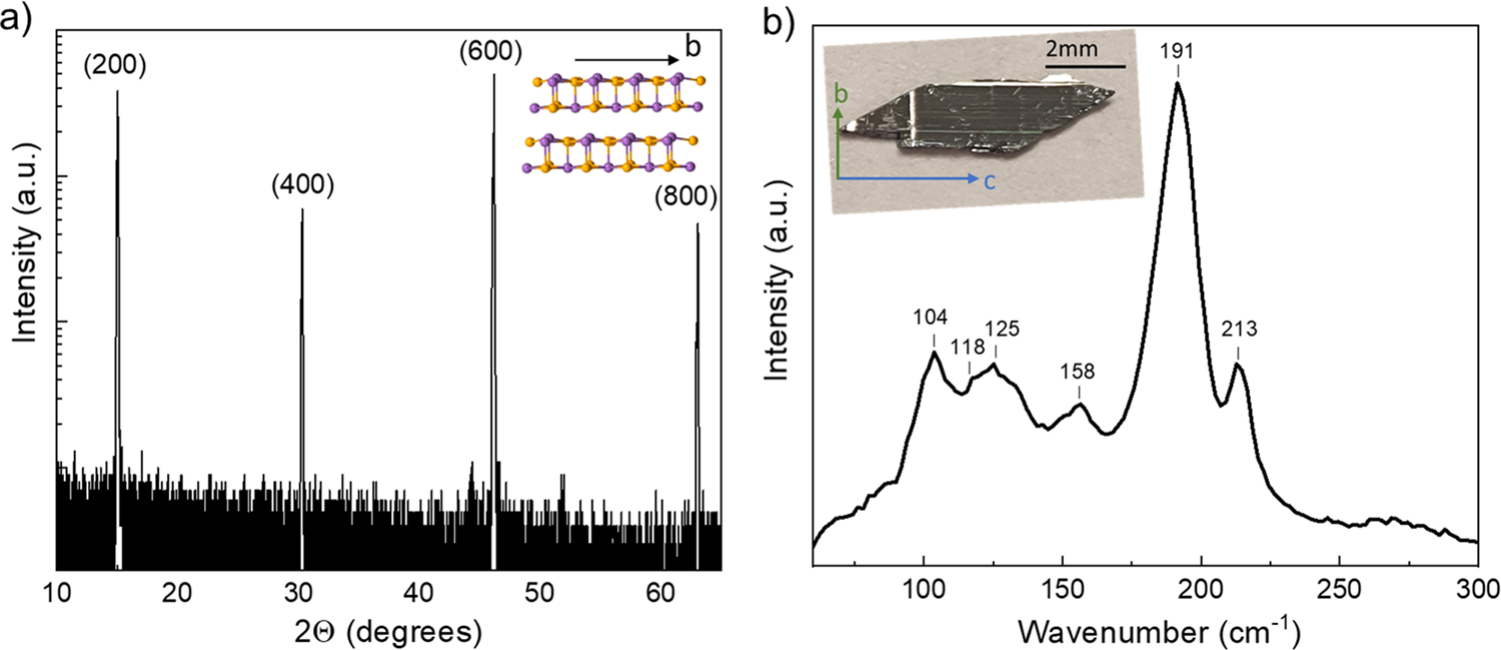
(a) XRD pattern of the annealed Sb_2_Se_3_ single crystal showing (100) surface orientation. Inset: a cut-out of the Sb_2_Se_3_ crystal structure highlighting a ribbon-like pattern. (b) Raman scattering spectrum of the annealed Sb_2_Se_3_ single crystal under 532 nm excitation wavelength. Inset: optical photograph of the annealed Sb_2_Se_3_ single crystal. Arrows indicate crystallographic axes.

The obtained sample was cut into smaller pieces with a diamond saw. Crystal planes (100) and (101) were the easiest to cleave. After cleaving, crystals had a highly reflective surface and geometrical area in 10–14 mm^2^ range (Figure 1b, inset). Multiple pieces were studied, and results were reproducible. However, in this study, we focus on Sb_2_Se_3_ single crystal samples, which were annealed under a sulfur + selenium atmosphere at 340 °C for 1 h. Annealing improved PL and PR responses. All data provided below are based on the annealed samples unless stated otherwise.

PL and PR measurements were recorded using a custom-made setup, which is depicted in Figure S2 of the Supporting Information. PL was excited using a diode-pumped solid-state 532 nm CW laser. The laser beam was unfocused to avoid damaging the sample. The power density was controlled with neutral density filters in 8–110 W/cm^2^ range. PL was dispersed by using the 500 mm focal length monochromator (Andor SR-500i; grating 600 g/mm blazing at 1000 nm) and focused into a thermoelectrically cooled InGaAs photodetector (IGA-030-TE2-H; Electro-Optical Systems Inc.). The conventional lock-in detection system (SR830; Stanford Research Systems) was used to extract the emission signal. The PL measurements were carried out in the temperature range of 3–160 K by mounting the samples on the cold finger of a closed-cycle helium cryostat (Janis SHI-4; Lake Shore Cryotronics, Inc.).

Temperature-dependent PR spectra were measured using a mechanically modulated (∼195 Hz) 532 nm laser light (modulation source) and a 100 W tungsten-halogen lamp (probe source). A double-monochromator system was utilized of which more details can be found in refs (18, 19).

The polarization-dependent PR signal was obtained using a polarization module POL (see the top inset in Figure S2 in the Supporting Information), consisting of λ/2 waveplate (rotated) and Glan-Taylor prism (fixed). The λ/2 waveplate was rotated between 0° and 45°, allowing changing the state of linear polarization from 0° to 90°. Such a configuration enabled investigating the polarized optical response from the mutually perpendicular crystallographic axes *b* and *c* in the Sb_2_Se_3_ single crystal. The *c*-axis of the sample and Glan-Taylor prism were intentionally rotated by 45° with respect to normal of the optical table, taking into account the polarizing property of the diffraction grating in a monochromator.

A Rigaku diffractometer SmartLab equipped with a 9 kW rotating Cu anode was used in identifying the crystalline facet of a Sb_2_Se_3_ single crystal. A double germanium monochromator (Ge(400) × 2) was added to get rid of the CuK_α2_ line. The sample was measured using θ–2θ geometry in the 10–68° 2θ angle range with a step size of 0.0052°.

Raman measurements under 532 nm wavelength excitation were performed by using an inVia Raman microscope (Renishaw, Wotton-under Edge, UK) equipped with the 1800 lines/mm grating and thermoelectrically cooled (−70 °C) CCD camera. The Raman spectrum was taken using a long working distance 50×/0.50 NA (Leica) objective lens. Laser power was restricted to 0.45 mW, and the integration time was set to 150 s. The Raman frequencies were calibrated using the silicon standard according to the line at 520.7 cm^–1^.

Compositional analysis was carried out in scanning electron microscope HELIOS Nanolab 650 (FEI) arranged with an X-ray energy-dispersive spectrometer (EDS) from Oxford Instruments. Impurity concentration in Sb_2_Se_3_ single crystals was estimated by inductively coupled plasma optical emission spectrometer (ICP-OES) Optima 7000DV.

## Results

X-ray diffraction (XRD) peaks’ positions matched very well with plane (*h*00), where *h* = 2, 4, 6, 8 position from PDF card no. 01-075-1462. Therefore, the exposed crystal plane of Sb_2_Se_3_ was (100) considering the *Pnma* space group, where b is the ribbon axis of Sb_2_Se_3_ (Figure 1a). Following other studies, we also found that the ribbon direction on the crystal was perpendicular to the long axis of the monocrystal (Figure 1b, inset). The Raman scattering spectrum was measured under 532 nm wavelength excitation to confirm phase purity and the absence of elemental Se or S aggregation on the surface after annealing (Figure 1b). All Raman bands matched well with previously published studies on Sb_2_Se_3_ single and polycrystals,^20,21^ and no evidence of secondary phases was seen. The chemical composition of samples was measured by EDS. Five points with an area of 400 × 400 μm^2^ spread uniformly over the samples were measured at 20 kV accelerating voltage. Before the measurements, EDS detector intensity was calibrated using a Cu piece. After the annealing, the composition was found to be slightly Sb-rich with a very small quantity of S. Atomic composition was as follows: Sb-41.5 ± 0.2, Se-58.2 ± 0.1; S-0.4 ± 0.1 at %. Although the estimated content of S was close to the sensitivity limit of the spectrometer, an evident peak of S K_α1_ in the spectrum was observed (Figure S3, Supporting Information).

CPs in the electronic band structure of Sb_2_Se_3_ crystals were studied using PR spectroscopy. PR spectra were fitted using third-order derivative lineshape following Aspnes complex function formulation:^22^
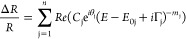
1where Δ*R*/*R* represents normalized change in reflectance; *C* represents amplitude, θ represents phase, *E*_0_ represents critical point energy position, Γ represents the broadening parameter, and *m* represents the parameter depending on the CP nature.

In total, three CPs were identified and were assigned as CP-α, CP-β, and CP-γ in order of increasing energy. To clearly showcase and distinguish all the features in the PR spectrum, we rely on the results obtained from three samples from the same batch: **sample A**—annealed Sb_2_Se_3_ as described in the Experimental Section, where PR was observed up to 100 K and was used for temperature-dependent PR analysis; **sample B**—annealed the same ways as sample A, but had a more pronounced CP-α contribution; (3) **sample C**—not annealed Sb_2_Se_3_ crystal, featuring only CP-α and was used to describe the shape of the CP-α spectrum.

**Figure 2:**
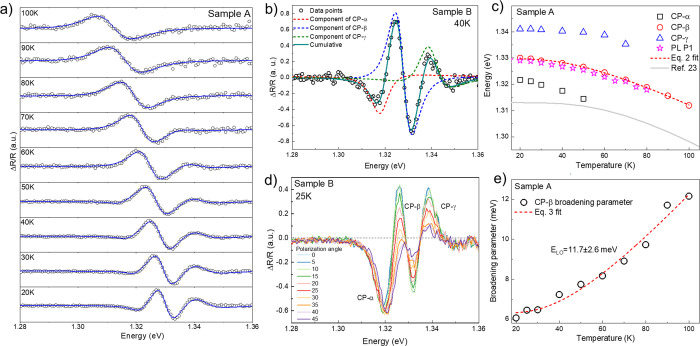
(a) Unpolarized temperature-dependent PR spectra of Sb_2_Se_3_ crystal sample A. The solid blue line indicates Aspnes fit eq 1. (b) Deconvoluted PR spectra of the Sb_2_Se_3_ crystal (sample B) measured at 40 K temperature. (c) CPs’ and PL P1 energy positions as a function of temperature in sample A. Red dotted lines show Bose-Einstein fit (eq 2). A gray solid line was calculated using parameters from ref (23). (d) Polarization angle-dependent PR spectra of the Sb_2_Se_3_ crystal measured at 25 K in sample B. (e) CP-β PR spectra linewidth as a function of temperature in sample A.

CPs started to appear as the temperature was decreased below 100 K, and contributions of CP-α and CP-γ were distinguishable at 50 and 70 K, respectively (Figure 2a). Their deconvolution at 40 K of sample B is presented in Figure 2b. To keep fitting results consistent, the phase factor (θ) in eq 1, which contributes to the shape of the PR spectrum, was fixed at constant values derived at 80 and 60 K for CP-β and CP-γ, accordingly. Parameter *m* (except for CP-α) was fixed at 2.5, which represented the interband transition in the 3D band structure. While PR spectra of CP-β and CP-γ had a typical shape with positive and negative extrema, the PR spectrum of CP-α had an uncharacteristic peak-like form. Fitting parameters describing the PR spectrum shape of CP-α were derived first to avoid the erroneous contribution of the CP-α component to other spectrum elements. It was achieved by fitting the PR spectrum of the untreated Sb_2_Se_3_ sample where only CP-α was observed (Figure S4a, Supporting Information). To ascertain that both samples showed the same PR signature of CP-α, PR spectra were overlayed, and a very good match of low energy PR features was found (Figure S4b, Supporting Information). Note that if unrestricted in the fitting process of CP-α, parameter *m* varied in 1.3–1.35 range which does not carry physical meaning but allowed to fit CP-β and CP-γ consistently and provided information about the CP-α energy position. The uncharacteristic shape of CP-α could be the result of very closely spaced PR spectra with opposite amplitudes or phases, however impossible to deconvolute. A similar shape, in fact, was observed for polycrystalline Sb_2_Se_3_ thin films^23^ and for ion beam-synthesized FeSi_2_.^24^

Temperature-dependent energy positions of all CPs are presented in Figure 2c. CP-γ had the highest energy and a similar temperature dependent trend to CP-β. CP-α was of the lowest energy and with the quickest redshift upon temperature increase. Its trend was the same as CP-α measured in the Sb_2_Se_3_ sample C (Figure S4c, Supporting Information). Because of the lack of data points, only CP-β was fitted using the Bose–Einstein model:^25^
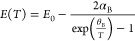
2where *E*_0_ represents energy at *T* = 0 K, α_B_ represents average electron–phonon interaction strength, and θ_B_ represents average phonon temperature. Calculated parameters are listed in Table 1.

**Table 1:** List of Calculated Fitting Parameters Based on Energy and Broadening Temperature Dependence of Sample A^a^^,^^b^^,^^c^^,^^d^^,^^e^^,^^f^

model	fitting parameters			
bandgap (*T*) equation 2	*E*_0_, eV	α_B_, meV/K	θ_B_, K	*E*@300 K, eV
	1.3297 ± 0.0001	0.033 ± 0.003	155 ± 7	1.233
gamma (*T*) equation 3	Γ_0_, meV	Γ_LO_, meV	θ_LO_, K	Γ@300 K, meV
	6.3 ± 0.2	17 ± 7	136 ± 23	36

a *E*_0_/Γ_0_, energy/broadening parameter at 0 K.

b α_B_, average electron–phonon interaction strength.

c θ_B_, average phonon temperature/energy.

d Γ_LO_, electron-longitudinal optical phonon coupling constant.

e Θ_LO_, longitudinal optical phonon temperature.

f Parameters are also calculated for the room-temperature case.

Average electron–phonon interaction strength and average phonon temperature were quite different from values obtained by other authors,^23,26^ implying the different origin of CP under study. The large difference in the absolute energy value also pointed to the different nature of CPs, although the overall temperature-dependent trend was similar above 60 K (Figure 2c). These discrepancies could be related to the different crystalline nature of the studied samples as well. Longitudinal optical (LO) phonon energy was estimated from the temperature-dependent broadening parameter of the CP-β PR spectrum (Figure 2e) according to the relation:^27^
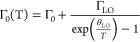
3where Γ_0_ is the broadening parameter at 0 K, Γ_LO_ is an electron-LO phonon coupling constant, and θ_LO_ is the LO phonon temperature. The latter value converted to energy was found to be 11.7 ± 2.6 meV (136 K) and, as will be discussed later, was consistent with PL results regarding excitonic line broadening.

Initially, the origin of observed CPs was tentatively ascribed to interband transitions (to different valence bands), but combining PL and PR results, we found that the CP-β energy position overlapped with excitonic PL peak (Figure 2c), suggesting that CP-β was of excitonic nature. Furthermore, higher in energy CP-γ had a similar temperature trend to CP-β, indicating that CP-γ should be related to the direct interband transition. Polarization-dependent PR measurements were conducted to identify the origin of CP-α, CP-β, and CP-γ. The intensity of the PR spectrum of CP-β and CP-γ was gradually and simultaneously decreasing with changing polarization angle from 0° to 45° which reflected a gradual wave vector shift from *E* ⊥ b (0°) to *E* ∥ b (45°) (Figure 2d). Such behavior confirmed that CP-β and CP-γ originated from the same valence band. Therefore, CP-β was related to the direct exciton, whereas CP-γ to the interband transition. This way the exciton binding energy could be estimated considering *E*_bX_ = *E*_CP-γ_ – *E*_CP-β_ and was found to be 13 meV on average over the measured temperature range. In fact, this value is in very good agreement with the activation energy calculated from temperature-dependent PL which will be discussed later.

On the other hand, the intensity of the PR spectrum of CP-α was almost independent of the polarization angle (Figure 2d). Polarization angle invariance of the CP-α PR spectrum suggested that transition related to CP-α does not consist of the valence band and that the defect state is involved. PR spectroscopy can indeed detect CP structures involving defects and is often applied to study defect formation.^28^ Based on the measured CP-α features, we propose that CP-α reflects the transition from the conduction band to the acceptor level, or even multiple closely spaced acceptor levels, therefore, leading to an uncharacteristic PR spectrum shape. Because at 3 K the energy difference between CP-α and CP-γ was small, the defect level involved in the CP-α transition was a shallow defect with ionization energy (*E*_i_) < 20 meV. Acceptor-type defects probably originated from impurity rather than from intrinsic Sb_2_Se_3_ point defects.^29^ More details about the nature of impurities will be discussed in the PL section.

Based on the results presented above, three CPs were identified in the Sb_2_Se_3_ single crystal. Optical features of CP-β and CP-γ were associated with direct bandgap free exciton and direct interband transitions, respectively, whereas CP-α with defect-related transitions.

PL of Sb_2_Se_3_ was excited using a CW laser operating at 532 nm wavelength. We note that care should be taken when choosing the excitation power density (especially under CW mode) because Sb_2_Se_3_ quickly heats up, leading to the decrease in PL intensity and unintentional redshift. In the extreme case, laser-induced decomposition of Sb_2_Se_3_ can occur which was stressed by other authors.^20^ Therefore, temperature-dependent measurements below 20 K were recorded under 8 W/cm^2^ and power-dependent—up to 50 W/cm^2^ excitation power density. No negative effects were observed even when excitation power density was increased up to 110 W/cm^2^ which was used to obtain higher PL intensity above 20 K.

**Figure 3:**
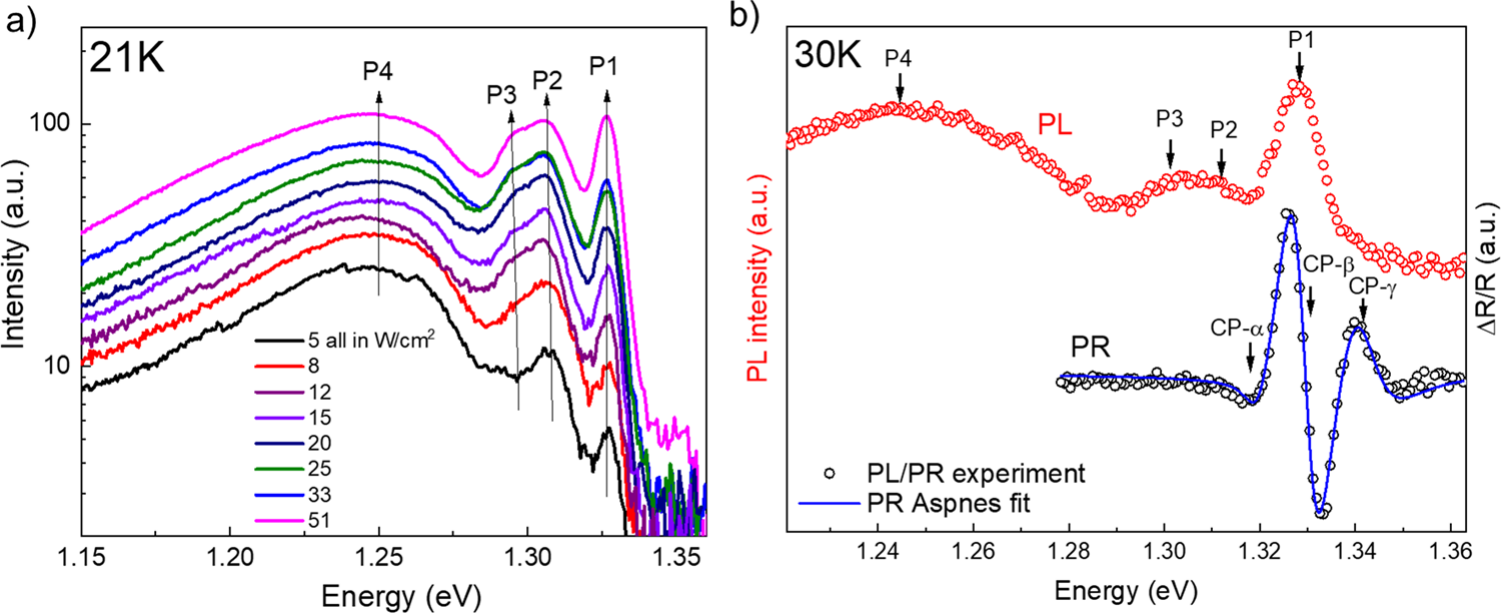
(a) Excitation power-dependent PL spectra of the annealed Sb_2_Se_3_ single crystal (sample A) at 21 K. (b) PL spectrum recorded at 30 K and under 51 W/cm^2^ excitation power density together with PR spectrum at 30 K of the annealed Sb_2_Se_3_ sample A.

Regardless of the crystal piece chosen from the batch, we always found that the PL spectrum of Sb_2_Se_3_ at low temperature consisted of many overlapping peaks. We focus first on the power-dependent PL spectra analysis measured at 21 K to identify emission characteristics. At this particular temperature, overlap of PL peaks was less pronounced, and several peaks could be resolved clearly. Four peaks labeled as P1, P2, P3, and P4 (in the order of decrease in energy) were identified observing power-dependent PL evolution (Figure 3a). The appearance of P3 in the form of the shoulder at the lower energy side of P2 became noticeable when excitation power density was higher than 10 W/cm^2^. P1 had a symmetrical Gaussian shape, P2 and P3 were overlapping hence difficult to distinguish, whereas P4 had a broad asymmetric form. Neither of the peaks showed an obvious energy position shift in relation to excitation power density. Note that the P1 position overlapped with the energy position of CP-β estimated from the PR measurements (Figure 3b).

**Figure 4:**
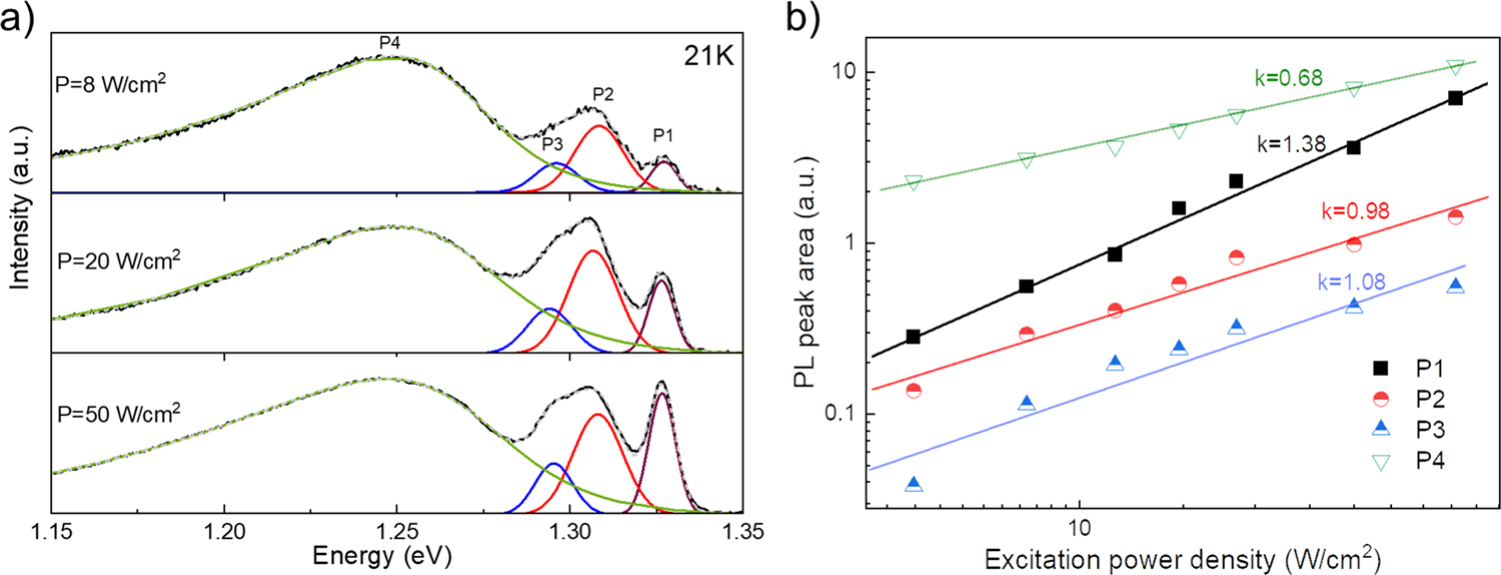
(a) Deconvoluted PL spectra of the annealed Sb_2_Se_3_ crystal (sample A) at three different power densities. Temperature was 21 K. (b) PL area dependence on excitation power density in a log–log scale.

Because of the high background and broad nature of PL peaks, it was not possible to define peak lineshapes precisely, but P1 and P2 were best described using the Gaussian function, whereas P4 was fitted using a double asymmetric sigmoidal function.^30^ For P3, also the Gaussian function was tentatively chosen. Deconvoluted PL spectra at representative excitation power densities are presented in Figure 4a. In the graph of integral PL area versus excitation power density, a good linear relationship was found for well-resolved peaks (P1 and P4), whereas P2 and P3 had a more scattered correlation (Figure 4b). According to the relation *I*_PL_ ∼ *P^k^*, where *I*_PL_ represents the integral PL area, *P* represents laser excitation power density, and *k* represents power coefficient, the calculated *k* values were 1.38, 0.98, 1.08, and 0.68 for P1, P2, P3, and P4, respectively. Obtained *k* values fall in three categories (*k* > 1, *k* ∼ 1, *k* < 1) which suggested that PL spectra consisted of emissions from three different radiative recombination mechanisms.^31^

**Figure 5:**
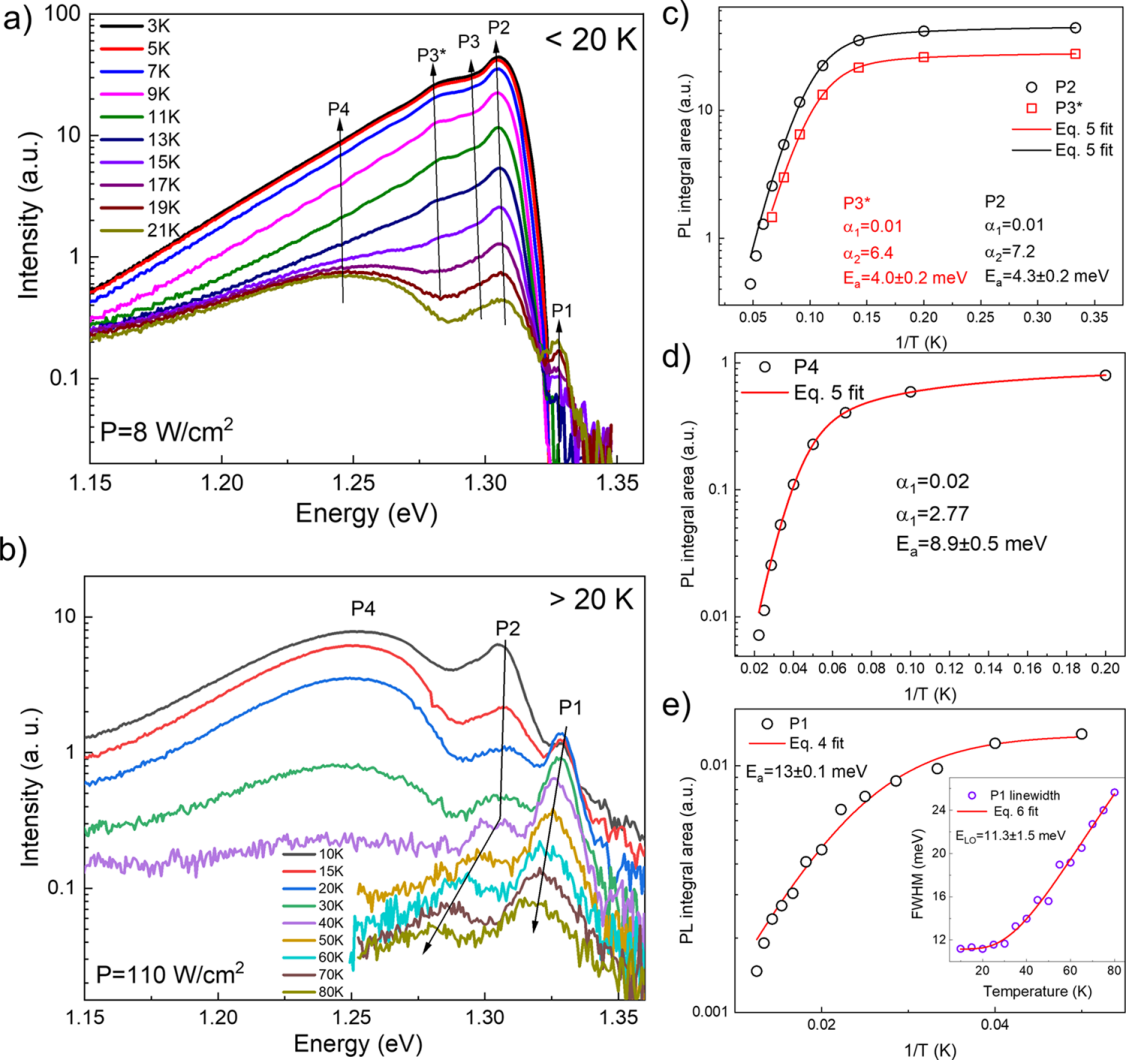
Temperature-dependent PL spectra of the annealed Sb_2_Se_3_ crystal (sample A): (a) below 20 K and under *P* = 8 W/cm^2^ and (b) above 20 K under *P* = 110 W/cm^2^ excitation power density. PL peak integral area as a function of reciprocal temperature: (c) P2 and P3*, (d) P4 and (e) P1. Inset in (e) FWHM dependence on the temperature for the peak P1. Solid lines show theoretical fits using indicated equations.

Based on the observed PL peak temperature sensitivity, PL analysis was divided into two cases: <20 and >20 K. Because of the very fast thermal quenching of peaks P2, P3, and P4, PL measurements below 20 K were performed at smaller temperature steps (Figure 5a). Upon a decrease in temperature, P1 started to decrease in intensity and became absent below 15 K, whereas the intensity of P2 and P3 increased significantly. Note that below 15 K a new PL peak located at ∼1.28 eV emerged (labeled P3*, Figure 5a). In addition, the exponential tail at the low-energy side comprised a substantial part of the PL spectra. We speculate that the origin of such a complex spectra resulted from the overlap of closely spaced and broad PL emissions, their phonon replicas, and phonon wings. As previously mentioned, LO phonon energy estimated from the temperature-dependent broadening parameter was around 12 meV (Table 1), which is comparable with the full width at half maximum (FWHM) of P1 and P2 even at 5 K. Therefore, it is very difficult if not impossible to deconvolute PL spectra and calculate their respective temperature-dependent positions and areas accurately. Therefore, assuming that the background was constant or constantly changing with temperature, we estimated temperature-dependent intensities by solely measuring their intensity at the maximum point of P2 and P3* peaks. To calculate thermally activated nonradiative recombination energy (*E*_a_) the following Arrhenius-type equations were considered: (i) single rate constant expression:
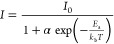
4and (ii) expression including two rate constants:^32^
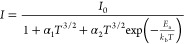
5where *I* represents PL intensity, *I*_0_ represents PL intensity near 0 K, α represents rate constant(s), *E*_a_ represents activation energy, *k*_b_ represents Boltzmann constant, and *T* represents temperature.

A poor fit was found using a single rate constant, whereas based on the second expression, a much better fit was obtained as evidenced from the calculated values of reduced χ2 (Figure S4d, Supporting Information). The calculated activation energy for both peaks P2 and P3* was very similar – about 4 meV (Figure 5c), indicating that they were of the same origin as expected from the excitation evolution shown in Figure 3a.

Above 20 K temperature, the PL peaks quenched fast, but P1 could be detected up to 80 K (Figure 5b). Moreover, P1 position dependence on the temperature was the same as CP-β within a few meV range (Figure 2c). Although a low-intensity (hardly resolvable) peak could be discerned close to the P2 position, we believe that after 40 K a stark shift of P2 is an indication of a new peak of another origin. However, it could not be analyzed in more detail because of low intensity. Peak P4 became absent above 40 K. The thermal activation energy of P4 and P1 was calculated using eqs 5 and 4, respectively (Figure 5d,e). We found that *E*_a_ for P1 was 13 ± 0.1 meV, whereas for P4 the fitting yielded *E*_a_ = 8.9 ± 0.5 meV. Temperature-dependent FWHM for peak P1 was fitted according to the expression:^33^
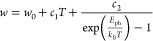
6where *w*_0_ is FWHM at 0 K, *c*_1_ is exciton-acoustic phonon scattering constant, *c*_2_ is exciton-optical phonon interaction constant, and *E*_ph_ is LO phonon energy (Figure 5e, inset). Calculated values were as follows: *c*_1_ = 1 μeV/K, *c*_2_ = 73 meV, and *E*_ph_ = 11.3 meV. Note that obtained LO phonon energy is in good agreement with PR results which estimated *E*_LO_ = 11.7 meV (see Figure 2e).

## Discussion

P1 had a symmetric shape, and the power-dependent coefficient was *k =* 1.38. P1 did not shift upon an increase of excitation power, started to appear when the intensity of P2 was decreasing and followed the direct bandgap temperature-dependent path, as shown in Figure 2c. Considering these characteristics, P1 was assigned to the free exciton (FE) emission. The binding energy, *E*_b_, of ground state FE can be estimated according to *E*_b_^X^ = *E*_g_ – *E*_x_. Based on the given PR study, we found that FE *E*_b_^X^ = 13 meV, which is in perfect agreement with the thermal quenching results for P1 matching the activation energy *E*_a_ = 13 meV (Figure 5e). Ground-state exciton binding energy, *E*_b_^X^, can also be calculated assuming the *hydrogenic* model as follows: *E*_b_ = μ*R*_y_/ε_0_^2^, where μ represents exciton reduced mass μ = *m*_e_^*^m_h_^*^/(*m*_e_^*^ + *m*_h_^*^), *R*_y_ is Rydberg constant (13.6 eV), ε_0_ – static dielectric constant, and m*_e/h_ represents electron/hole effective masses.^34^ Carrier effective masses have not been measured directly, but a recent theoretical study calculated m*_e/h_ and ε_0_ in Sb_2_Se_3_ and Sb_2_S_3_ along three principal directions.^35^ Their calculated material parameters agree well with the ones determined experimentally, signifying their reasonably accurate description of Sb_2_Se_3_ physical properties. Therefore, we used these values to calculate exciton binding energy along the Γ–*Z* direction where electronic band extrema are located. Calculated exciton *E*_b_ in the Γ–*Z* direction was extremely small (0.27 meV) because of the very large ε_x0_ (ε_x0_ = 128) leading to an effective screening of charged carriers. However, because of the quasi-one-dimensional crystal structure, Sb_2_Se_3_ physical properties are strongly anisotropic. To account for anisotropic effects, as a first approximation instead of ε_x0_, an average ε_0_ defined as  and mean values of effective carrier masses were taken. In this case, we found that exciton binding energy *E*_b_ was 1.5 meV, which is still almost 10 times lower than that estimated experimentally. This demonstrates that a simple *hydrogenic* model is not suitable for calculating exciton *E*_b_ of low dimensional materials. It has been shown that exciton *E*_b_ is larger than that expected in other layered compounds,^36^ which is also the case for Sb_2_Se_3_.

P2 quenched extremely quickly with temperature, which is one of the main characteristics of bound excitons (BE). In addition, the appearance of FE upon decrease of P2 intensity with temperature supports the idea of BE dissociating into FE. Such a case was also observed in other materials featuring FE and BE PL emissions simultaneously.^37^ The position of P2 was located at 1.305 eV at 3 K; therefore assuming that P2 is a direct bandgap BE, the binding energy, that is, the energy difference between P1 (FE) and P2 was around 25 meV. This unusually high binding energy for BE does not correlate with thermal activation energy (*E*_a_ = 4 meV) determined from temperature-dependent measurements (Figure 5c).

While P1 was confirmed to be direct bandgap FE transition, the P2 can be related to the indirect gap transition. Based on experiments^17^ and modeling,^38^ Sb_2_Se_3_ is an indirect bandgap semiconductor, and at very low temperatures, carriers will occupy the lowest energy states, which can lead to the formation of indirect BE.^39^ In this case, *E*_b_^BE^ = *E*_X_^ind^ – *E*_BE_, where *E*_b_^BE^ is the binding energy of BE, *E*_X_^ind^ is indirect bandgap free exciton energy, and *E*_BE_ is BE energy. Because *E*_X_^ind^ and its binding energy are unknown, we can only estimate the upper limit for *E*_b_^BE^. According to the theoretical modeling, indirect bandgap is about 10 meV smaller than the direct one at 0 K^38^ and assuming that binding energy of *E*_X_^ind^ is comparable with direct bandgap free exciton, we find that *E*_b_^BE^ < 12 meV. This value is more consistent with the activation energy estimated for P2; therefore, the origin of P2 is suggested to be emission from bound excitons within an indirect bandgap.

Furthermore, based on Zimmermann et al.,^40^ rapid quenching of BE emission is an indication that the neutral acceptor rather than the donor defect was involved in the formation of BE. Additionally, (A^0^,X) excitons feature a low-energy phonon wing and have strong coupling with LO phonons. A complex PL spectrum at the low-energy side of P2 including P3 and P3* thus originated from BE phonon wings and overlapping LO phonon replicas, as shown in Figure 5a.

To identify impurities that could be responsible for the formation of BE in the Sb_2_Se_3_ single crystal, we measured the concentration of the most probable contaminants found in precursor materials by ICP-OES. Among tested elements (Mn, Cr, Fe, Co, Cd, Cu, Pb, Zn, Sn, Bi, P, Zr), only the following two were detected: Cr – 3 ppm and P – 112 ppm. P seems to be a common unintentional impurity in antimony chalcogenides.^41^ Unfortunately, halides that are predicted to be shallow defects in Sb_2_Se_3_^42^ could not be tested because of limitations of the analysis method. Because P2 emission at 4 K temperature was observed in all Sb_2_Se_3_ single crystal samples and did not depend on the stoichiometry, it is suggested that BE must be associated with a commonly present impurity such as P instead of intrinsic defects. In a rough approximation, the ionization energy of defects involved in BE can be estimated using *E*_b_^BE^ as follows: for donor type *E*_b_^BE^ = 0.21 *E*_D_ and for acceptor *E*_b_^BE^ = 0.11 *E*_A_.^43^ Hence considering *E*_b_^BE^ = *E*_a_ (Figure 5c), the estimated ionization energy of the acceptor would lie in the 36–39 meV range. The concentration of these shallow acceptor defects is expected to be very low because the measured specific resistance of the samples was 5·10^7^ Ω·cm at room temperature. We see that the obtained acceptor ionization energy was almost twice as large as the one determined from PR results, suggesting that additional acceptor type impurities or defect levels in the bandgap were present.

P4 had an asymmetric and broad PL shape, and the center was located far from the absorption edge (1.245 eV) and had a power coefficient *k* of 0.68. All these features are characteristic of the emission from donor–acceptor pair (DAP) recombination and are in close agreement with the work of Grossberg et al.^15^ They investigated Sb_2_Se_3_ polycrystals and found that at low temperature (*T* < 25 K), the PL spectrum was dominated by asymmetric DAP emission located at 1.24 eV and had a power coefficient of 0.6. It implied that DAP emissions at ∼1.24 eV observed in the Sb_2_Se_3_ single crystal and polycrystals were of the same origin. Considering that Sb_2_Se_3_ indirect bandgap energy was 10 meV below the direct one (1.33 eV), defects involved in DAP emission had ionization energy below 80 meV. The authors did not specify defects responsible for DAP emission but suggested that Se_Sb_ antisite defects could be involved under Se-rich composition. Based on density functional theory calculations, only V_Sb_ (Sb vacancy) was predicted to be an acceptor type defect and have *E*_i_ < 100 meV when the Fermi level is positioned in the middle of *E*_g_.^44^ Keeping in mind that Grossberg et al. studied Sb_2_Se_3_ polycrystals that were Se-rich, whereas in this work Sb_2_Se_3_ single crystals were Sb-rich, which implied that defects involved in the same observed DAP recombination originated from common extrinsic rather than intrinsic point defects. Previous studies have shown the presence of unintentional donor type Cl impurity in the commercially available high-purity Sb_2_Se_3_ powder.^45^ Therefore, we propose that shallow defects forming DAP could be related to the P and halide (e.g., Cl) impurities commonly found in the antimony chalcogenide source materials.

However, to fully understand how impurities affect PL properties of Sb_2_Se_3_, characteristics of extrinsic defects and their possible defect complexes should be studied on the fundamental level. Notably, several extrinsic acceptor type defects such as Sn, Cu, Pb, and donor type such as Cl, Br, and I have been investigated via first-principles calculations.^42,46^ It was found that all potential *p*-type dopants are deep defects with *E*_i_ above 200 meV, whereas *n*-type are more shallow defects, *E*_i_ < 50 meV. Other impurities, for instance P that was present in our samples in a significant amount has not been studied. Additionally, defect complexes can also introduce a deep/shallow energy level in the bandgap and in turn can play an important role in electrical and optical characteristics. Therefore, relating observed PL emission to specific defects in Sb_2_Se_3_ requires a systematic study of various extrinsic defects and their complexes in Sb_2_Se_3_, which however is beyond the scope of the current work.

**Figure 6:**
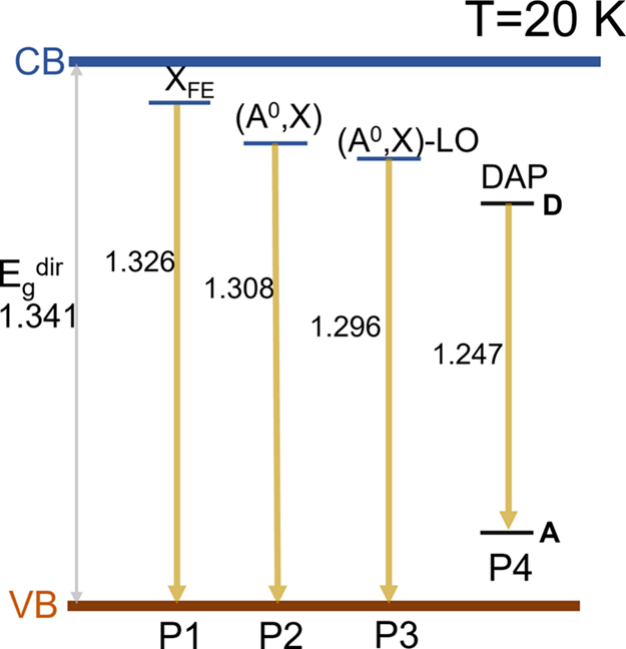
Schematic diagram of radiative recombination processes observed in Sb_2_Se_3_ single crystals at 20 K. All numbers are expressed in eV and indicate PL emission energy. Notations X_FE_, (A^0^,X), (A^0^,X) – LO, and DAP correspond to direct free exciton, bound indirect exciton, bound indirect exciton phonon-assisted, and DAP recombination mechanisms, respectively.

The summary of radiative recombination mechanisms observed in Sb_2_Se_3_ single crystals in the 3–80 K temperature range is presented in Figure 6. Overall, excitonic emission dominated PL spectra. Below 20 K temperature, PL emission mainly occurred from bound excitons (A^0^,X), their phonon replicas and DAP recombination. Above 20 K temperature, the main contribution to PL emission was from radiative recombination of free excitons and was observable up to 80 K.

## Conclusions

In conclusion, PR and PL studies revealed a complex nature of the Sb_2_Se_3_ electronic structure and optical transitions. Nonintentional impurities played the main role in the low-temperature PL characteristics. Free exciton, bound exciton, and DAP optical transitions were identified in the PL spectrum. However, no emissions were observed from self-trapped excitons or band-tails that could fundamentally put the upper limit for open-circuit voltage in Sb_2_Se_3_-based solar cells. On the other hand, fast thermal quenching and low PL intensity signified considerable nonradiative recombination likely through deep defects, which is the primary source of the high *V*_OC_ deficit.

## Supporting Information Available

Absoprtion and PL spectra of Sb_2_S_3_; schematic representation of the PL and PR measurement setup; EDS spectrum of the annealed Sb_2_Se_3_ sample; PR spectra of samples B and C; and CP energy and PL intensity as a function of temperature (PDF)
