# Supplementary material for: Photoreflectance and Photoluminescence Study of Antimony Selenide Crystals
Source: ACS Appl Energy Mater. 2022 Nov 16;5(12):14769–78. doi: 10.1021/acsaem.2c02131 (PMC9795641; doi:10.1021/acsaem.2c02131)
Supplement: Supplementary file 1 — ae2c02131_si_001.pdf [file ae2c02131_si_001.pdf]

SUPPORTING INFORMATION

Photoreflectance and photoluminescence study of antimony selenide crystals

Rokas Kondrotas*[a], Ramūnas Nedzinskas[a], Jüri Krustok[b], Maarja Grossberg[b], Martynas Talaikis[a], Saulius Tumėnas[a], Artūras Suchodolskis[a], Raimundas Žaltauskas[c], Raimundas Sereika[c]

[a]*State Research Institute, Center for Physical Sciences and Technology, Saulėtekio Ave. 3, Vilnius, 10257, Lithuania*
[b]*Department of Materials and Environmental Technology, Tallinn University of Technology, Ehitajate Tee 5, 19086, Tallinn, Estonia*
[c]*Vytautas Magnus University, K. Donelaičio str. 58, 44248 Kaunas, Lithuania*

*Corresponding author email: rokas.kondrotas@ftmc.lt

Figure S1. (a) Absorption and PL spectra of Sb2S3 single crystal, reproduced from Yang et al [1] under CC BY license.

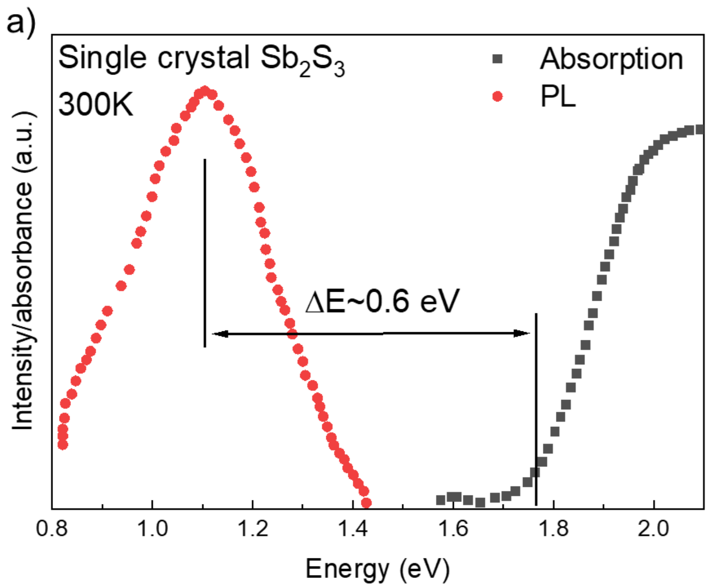

Figure S2. Schematic representation of custom-made PR and PL measurement setup.

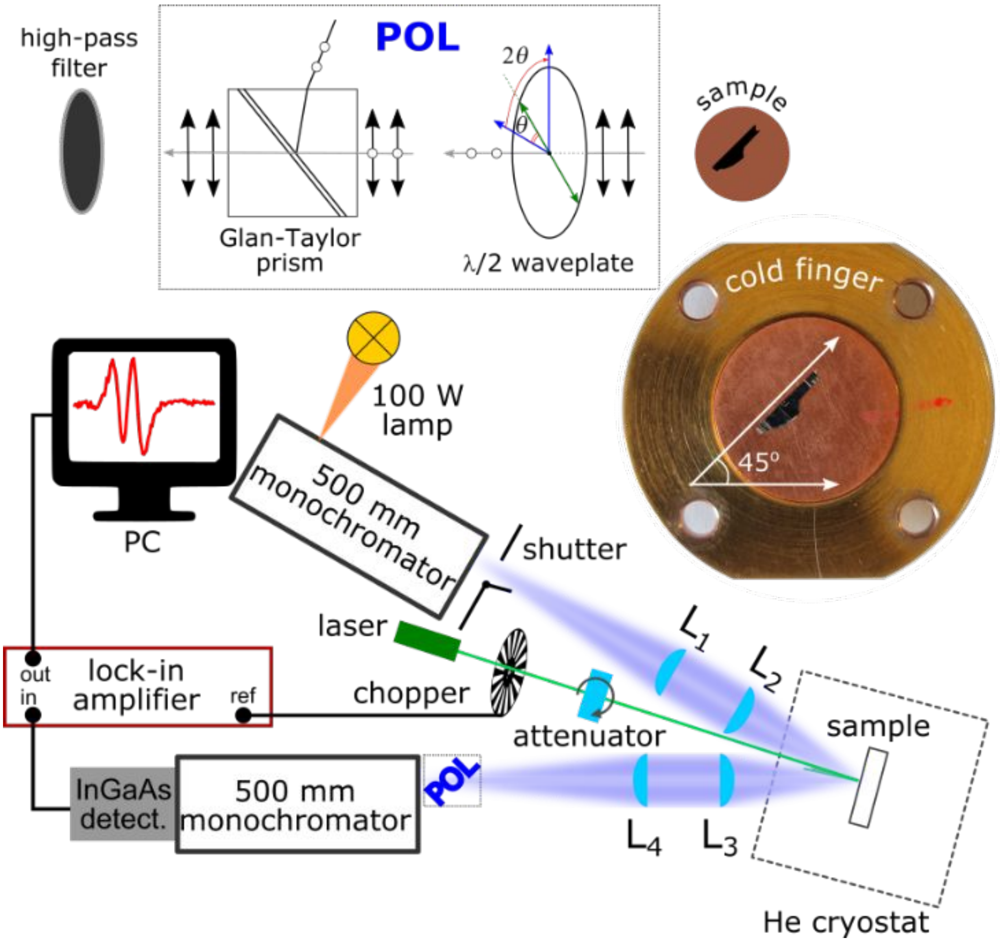

Figure S3. X-ray Energy dispersive spectrum of the annealed Sb_2Se_3 single crystal (sample A).

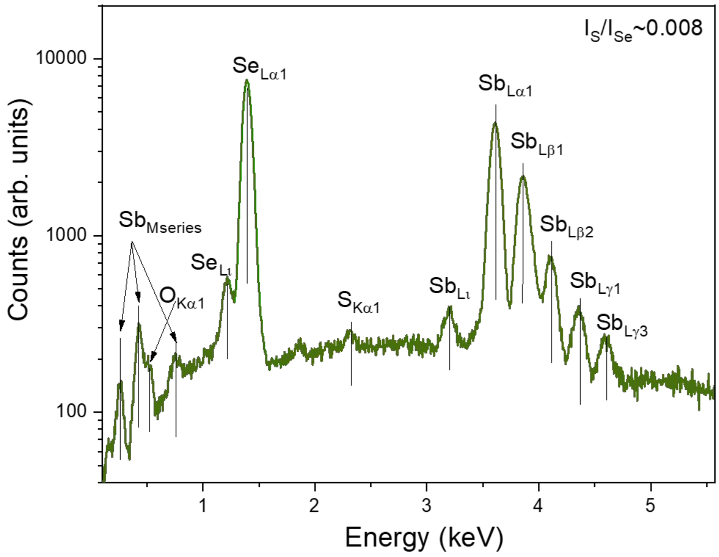

Figure S4. (a) PR spectra of sample C at different temperatures showing the presence of only CP-α. (b) PR spectra of sample B and C at 40 K showing the overlap of CP-α. In sample C, the PR signal was normalized to match amplitudes of CP-α in sample B. (c) Temperature-dependent CP energy positions of sample A and C. (d) Fitting of the temperature dependent PL intensity using one and two rate constants.

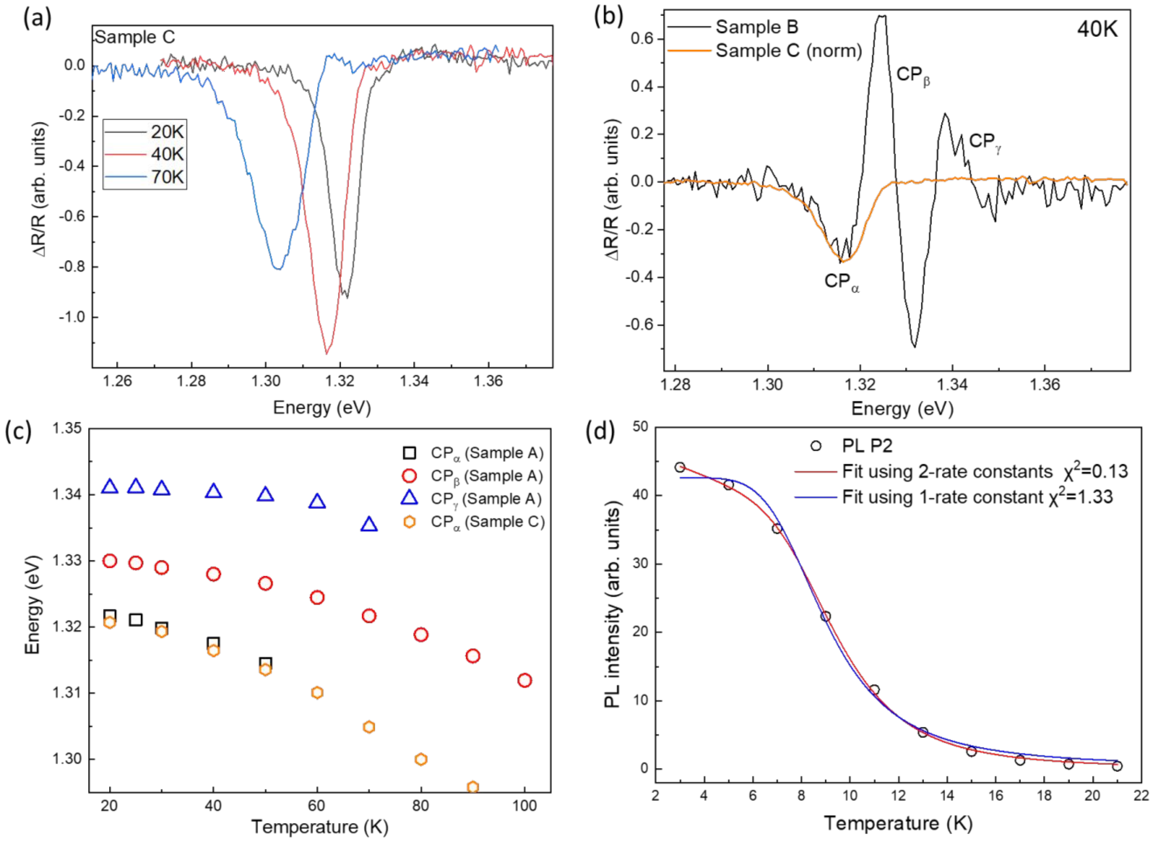

Reference:

1. Yang, Z.; Wang, X.; Chen, Y.; Zheng, Z.; Chen, Z.; Xu, W.; Liu, W.; Yang, Y. M.; Zhao, J.; Chen, T. Ultrafast self-trapping of photoexcited carriers sets the upper limit on antimony trisulfide photovoltaic devices. Nat. Commun. 2019, 10 (1), 1-8.
